# *Fusarium* Species and Mycotoxins Associated with Sorghum Grains in Uruguay

**DOI:** 10.3390/toxins15080484

**Published:** 2023-07-31

**Authors:** Ana Belén Corallo, Agustina del Palacio, María Oliver, Susana Tiscornia, Macarena Simoens, Jaqueline Cea, Inés de Aurrecoechea, Inés Martínez, Alicia Sanchez, Silvina Stewart, Dinorah Pan

**Affiliations:** 1 Sección Micología, Facultad de CienciasFacultad de Ingeniería, UdelaR, Julio Herrera y Reissig 565, Montevideo 11300, Uruguay; belencfabiano@gmail.com (A.B.C.); agustinadelpalacio@gmail.com (A.d.P.); moliver24@gmail.com (M.O.); tiscorniasusana@gmail.com (S.T.); 2 Laboratorio Tecnológico del Uruguay, Departamento de Análisis de Productos Agropecuarios, Avenida Italia 6201, Montevideo 11500, Uruguay; msimoens@latu.org.uy (M.S.); jacqueline.cea6@gmail.com (J.C.); 3 Departamento de Granos, Dirección General de Servicios Agrícolas, Ministerio de Ganadería Agricultura y Pesca, Avenida Millán 4703, Montevideo 12900, Uruguay; ideaurrecoechea@mgap.gub.uy; 4 Latitud, Fundación del Laboratorio Tecnológico del Uruguay, Avenida Italia 6201, Montevideo 11500, Uruguay; imartin@latitud.org.uy (I.M.); asanchez@latitud.org.uy (A.S.); 5 Programa Cultivos de Secano, Instituto Nacional de Investigación Agropecuaria, Estación Experimental La Estanzuela, Ruta 50, Km 11, Colonia 70000, Uruguay; sstewart@inia.org.uy

**Keywords:** *Fusarium graminearum*, *Fusarium fujikuroi* species complex, trichothecene, zearalenone, fumonisin, fungicide sensitivity

## Abstract

Grain mold and stalk rot are among the fungal diseases that cause significant losses in sorghum worldwide and are caused by different *Fusarium* spp. The presence of *Fusarium* species in sorghum grains causes yield losses and mycotoxin contamination, which represents a risk to consumers. In this study, *Fusarium graminearum* species complex (FGSC) had a high incidence, followed by *Fusarium fujikuroi* species complex (FFSC) and *F. incarnatum-equiseti* species complex. Within FFSC, *F. proliferatum*, *F. andiyazi*, *F. fujikuroi*, *F. thapsinum*, *F. verticillioides* and *F. subglutinans* were identified, and this was the first report of *F. fujikuroi* in sorghum. The most frequent toxins found in sorghum samples were deoxynivalenol (DON) and zearalenone (ZEN). The presence of fumonisins and nivalenol (NIV) was detected at low levels. This study adds new knowledge about the occurrence of *Fusarium* species and mycotoxins in sorghum grains. Furthermore, this is the first report in Uruguay on fungicide sensitivity for *Fusarium* isolates from sorghum, which constitutes an important starting point for defining management practices to minimize fungal infection and mycotoxin contamination.

## 1. Introduction

Sorghum (*Sorghum bicolour* L. Moench), with a world production of 62 × 10^6^ t in 2021, is one of the most important cereals [1,2].

With the discovery of sorghum’s associated health benefits, food-grade sorghum has been produced and is becoming more popular in different regions of the world as a human food [3]. These nutritional qualities, in addition to increasing the profitability of sorghum, have increased the total cultivated area worldwide [4].

In Uruguay, sorghum is one of the three major grain fed summer crops, and it is used for feeding cattle, dairy cattle, poultry, and swine. It´s is considered one of the main protagonists, both for its stability and for its low production cost, which contributes to increasing the profitability of the livestock and dairy production system. Stems and leaves are used for silage, and grains are destined for animal feeding or bioethanol production [5]. For this reason, the sorghum cultivated area has increased by 237% from 2000 to 2021, with 233 × 10^3^ t harvested in the 2020–2021 season [6].

Although sorghum is considered to have habitual resistance to microbial infection, fungal contamination is a major limitation for sorghum production [3]. Among the fungal diseases that causes significant losses in the world are grain mold and stalk rot, which are caused by different *Fusarium* spp. [7]. The presence of *Fusarium* species in sorghum grains causes yield losses and represents a risk to the health of consumers due to the capacity to produce mycotoxins, secondary toxic metabolites.

The *Fusarium fujikuroi* species complex (FFSC) is usually associated with sorghum in different countries [3,8,9,10,11,12]. In addition, some species of this complex can produce fumonisins (FBs), a group of mycotoxins responsible for the contamination of sorghum grains [13]. The presence of FBs in grains and feed has been associated with several diseases such as leukoencephalomalacia equine and porcine pulmonary edema. Further, human consumption has been epidemiologically associated with esophageal cancer and neural tube defects [14]. Other major *Fusarium* spp. associated with sorghum are the members of the *Fusarium graminearum* species complex (FGSC), which produce type B trichothecene mycotoxins such as deoxynivalenol (DON), nivalenol (NIV) and zearalenone (ZEN). DON is associated with feed refusal, vomiting, and suppressed immune functions, and ZEN can cause changes in reproductive organs and fertility loss in animals and humans due to the affinity for estrogen receptors [15,16].

Considering the above and the lack of information that exists in Uruguay, the aims of the present study were (1) to identify the *Fusarium* species associated with sorghum grains in the major sorghum-growing area in Uruguay, (2) to determine the presence of mycotoxins in sorghum samples, (3) to assess the ability of *Fusarium* spp. to produce mycotoxins, and (4) to evaluate the in vitro sensitivity of the main *Fusarium* species towards four fungicides corresponding to triazoles and benzimidazoles, currently used on several crops.

## 2. Results

### 2.1. Species Identification

A total of 1567 *Fusarium* spp. from the 2016 harvest and 1098 from the 2017 harvest were isolated in 95 and 72 sorghum grain samples, respectively.

**Figure 1:**
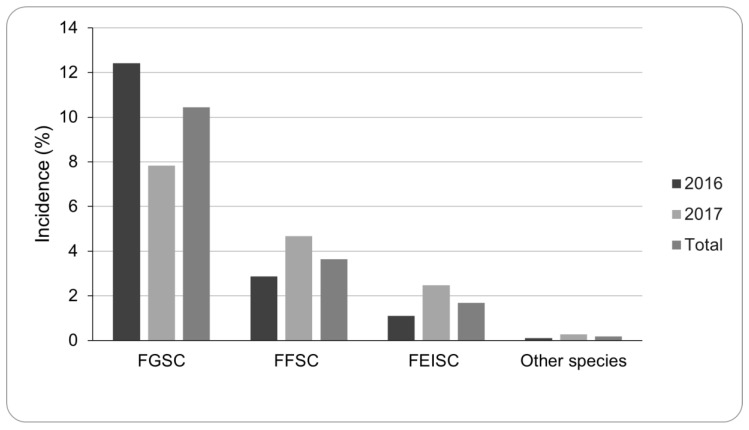
Incidence (%) of the different *Fusarium* species infecting the sorghum samples during 2016 and 2017 as assessed by visual observations. Incidence was calculated as the percentage of grain infected out of the total analyzed grains per sorghum sample. *Fusarium graminearum* species complex (FGSC); *Fusarium fujikuroi* species complex (FFSC); *Fusarium incarnatum-equiseti* species complex (FIESC).

In 2016, mean *Fusarium* spp. incidence in grain samples was 17%, with a maximum of 84%. During 2017, the incidence was lower (15%), although the maximum incidence was 100%. Among the *Fusarium* species isolated and based on morphological characteristics, FGSC had the highest incidence (10%, *n* = 1744), followed by species belonging to FFSC (4%, n = 609) and the *F. incarnatum*-*equiseti* species complex (FIESC) (2%, n = 282) (Figure 1).

Within FGSC, *F. graminearum* s.s was the predominant species, with only two isolates identified as *F. boothii*. Meanwhile, within the FFSC, 28% were identified as *F. proliferatum*, 23% as *F. andiyazi*, 21% as *F. fujikuroi*, 18% as *F. thapsinum*, 5% as *F. verticillioides* and 5% as *F. subglutinans* (Figure S1 Supplemental Material). Other identified species were *F. heterosporum* (2%), *F. tricinctum* (1%), *F. armeniacum* (1%), *F. pseudograminearum* (0.5%), *F. chlamydosporum* (0.5%) and *F. oxysporum* (0.2%).

### 2.2. Trichothecene Genotype Determination

The multiplex PCR assays indicated that most *F. graminearum s.s* isolates were of the 15AcDON genotype (98%), with only two isolates indicating NIV genotypes. No 3-AcDON genotypes were determined in the growing seasons studied.

### 2.3. Fusarium Mycotoxin Production

The mycotoxin production ability of *F. graminearum s.s* and *F. proliferatum* isolates was analyzed, as they were the most frequent species. Of the *F. graminearum s.s* studied, ZEN was produced by 92% (*n* = 86) of the isolates, DON by 88% (*n* = 83) and NIV by 6% (*n* = 6). Production of FB1 and FB2 was detected in 75% (*n* = 41) and 64% (*n* = 35) of the *F. proliferatum* isolates, respectively. Thirteen *F. proliferatum* isolates (24%) did not produce fumonisins.

### 2.4. Determination of Mycotoxins in Sorghum

One hundred twelve sorghum samples harvested during 2016 and 2017 were analyzed for the presence of mycotoxins (Table 1). The percentage of DON-positive samples was high during the two years (88% and 93%), while mean DON content was low, varying from 1315 μg/kg to 565 μg/kg, respectively. Regarding ZEN, positive samples (97 and 90%) and the mean content (1453 μg/kg and 752 μg/kg) were high in both years of study. Also, the annual maximum levels ranged from 6560 μg/kg to 9280 μg/kg. Low levels of FB1 and FB2 were also detected in the sorghum samples. Similarly, low levels of NIV were only detected in three samples from 2017.

### 2.5. Fungicide Sensitivity Assay

**Figure 2:**
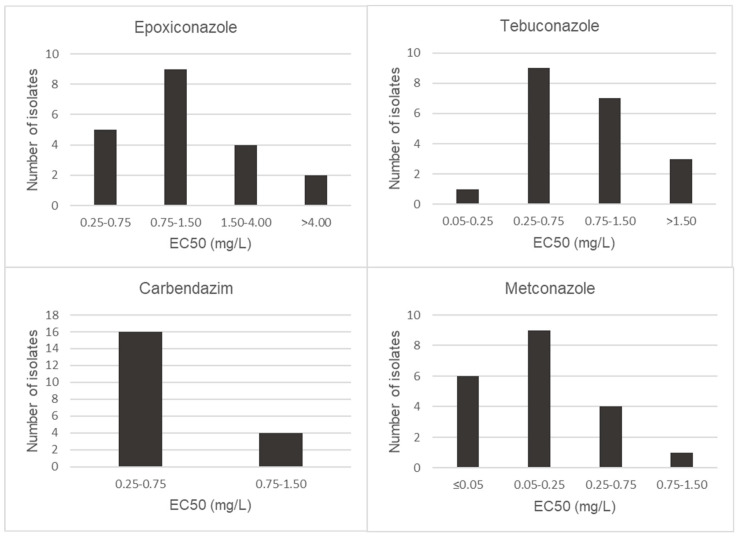
Distribution of EC50 (mg/L) values of *Fusarium proliferatum* isolates for epoxiconazole, tebuconazole, carbendazim and metconazole.

**Figure 3:**
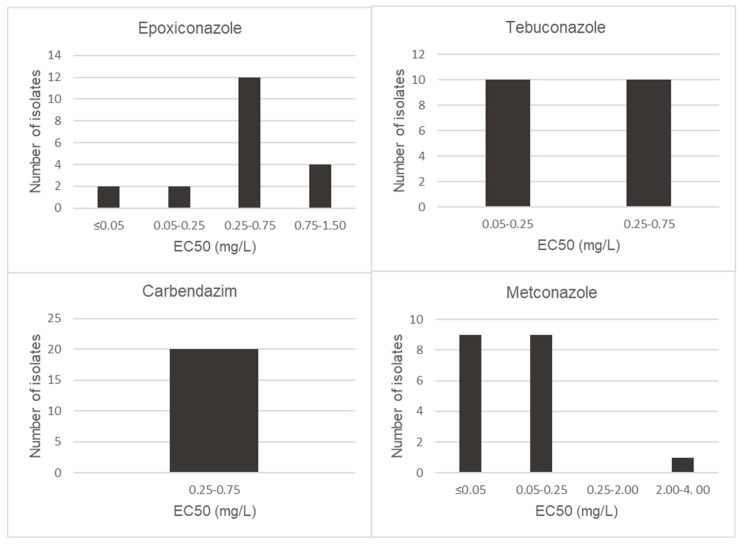
Distribution of EC50 (mg/L) values of *Fusarium graminearum s.s* isolates for epoxiconazole, tebuconazole, carbendazim and metconazole.

EC50 values of metconazole, tebuconazole, epoxiconazole and carbendazim were calculated for 20 isolates of *F. proliferatum* and *F. graminearum s.s.* Both *Fusarium* species showed different behaviors to the tested fungicides (Figure 2 and Figure 3). The two *Fusarium* species investigated showed the highest sensitivity levels for metconazole, with EC50 values below 0.50 mg/L and significantly different from those of the other fungicides tested (*p* < 0.05). Only one isolate of *F. proliferatum* was shown to be less sensitive to metconazole, with EC50 values of 2.18 mg/L. Besides metconazole, EC50 values lower than 0.75 mg/L were recorded for carbendazim and tebuconazole for all isolates of *F. proliferatum*, with mean EC50 values of 0.53 mg/L and 0.33 mg/L, respectively. In the case of epoxiconazole, isolates S400 and S677 were observed to be the least sensitive, with EC50 values of 0.99 and 1.04 mg/L, respectively.

Generally, isolates of *F. graminearum* were shown to be less sensitive to the fungicides evaluated. The EC50 values for triazole fungicide class ranged from 0.31 mg/L to 15.29 mg/L with a mean of 1.97 mg/L for epoxiconazole, 0.05 mg/L to 0.97 mg/L with a mean of 0.17 mg/L for metconazole and 0.14 mg/L to 3.54 mg/L with a mean of 1.06 mg/L for tebuconazole (Figure 3). Additionally, carbendazim EC50 values ranged between 0.34 mg/L and 1.37 mg/L with a mean of 0.62 mg/L (Figure 3). In the case of epoxiconazole, isolate S501 was observed to have high EC50 values of 15.29 mg/L. Also, isolates S58, S68 and S501 showed low levels of sensitivity for tebuconazole, with values of 2.76, 2.88 and 3.54 mg/L, respectively.

## 3. Discussion

In Uruguay, little is known about the presence of fungi and mycotoxins in sorghum. Most of the studies that have been carried out have focused on describing the occurrence and incidence of *Fusarium* species in wheat and barley [17,18,19]. Only one study was found for this crop, and it was limited to silages [20]. Considering this, this work represents the first extended study about the occurrence of *Fusarium* species in sorghum grain in Uruguay. *Fusarium graminearum s.s.* appeared as the predominant species, followed by *F. proliferatum*, *F. andiyazi*, *F. fujikuroi* and *F. thapsinum.* Although all of these species have previously been present in sorghum in other parts of the world, our data indicate that *F. graminearum s.s.* is the main contaminant of sorghum, which differs from that reported in other studies where FFSC resulted in the most frequent contaminants [8,9,10,11,21,22]. This can be partially explained by the crop rotation system in Uruguay, where sorghum is generally planted after wheat or barley, so the prevalence of *F. graminearum s.s* could be due to inoculum carryover from the previous crop. The presence of *F. graminearum* in sorghum grains has a great toxicological risk since this species can produce DON, 15-ADON, 3-ADON, NIV and ZEN. Also, infection of sorghum by species within FGSC can cause reduced germination and nutritional quality of the grains [23].

Within FFSC, *F. proliferatum*, *F. andiyazi* and *F. thapsinum* were the most frequent species, with all these species being reported for the first time in Uruguay. These species have been isolated from sorghum in Argentina, Australia, Brazil, Ethiopia, Nigeria, South Africa, Tunisia, and the USA and are well documented as pathogens of sorghum that cause grain mold and stalk rot [10,11,21,22,24,25,26]. However, frequencies of FFSC in this study were in contrast with species recovered from sorghum in other areas, including Argentina and Brazil, where *F. thapsinum* and *F. verticillioides* are the most frequent [8,11,12].

*Fusarium fujikuroi* was also recovered with high frequency within FFSC and, to our knowledge, this is the first report in sorghum. It has been isolated from rice, where it causes Bakanae disease, and from other crops such as soybeans, corn, barley, wheat, and peanuts.

These differences in the recovery and composition of FFSC may be associated with differences in agroclimatic and crop rotation systems associated with our country [26]. Moreover, little is known concerning the growth conditions, host range and pathogenicity of the *Fusarium* species associated with sorghum in Uruguay. This knowledge is essential for developing appropriate management strategies.

*Fusarium graminearum s.s* with the DON/15ADON genotype was the predominant species and represents the first data of *F. graminearum* s.s strains isolated from sorghum in Uruguay. Our results agree with previous reports from different crops in Uruguay and other regions of South America, where the DON-15ADON genotype prevails for *F. graminearum s.s.*, showing the stability of genotype composition across crops and years [17,27,28].

Mycotoxins in sorghum samples showed contamination with FB1 and FB2, but in low levels, and none of the samples exceeded the limit established by the FDA for animal feed and the current regulations for other grains [29]. Incidence and DON levels found in the present study were high when compared to other reports from sorghum [30,31]. However, only 27% (*n* = 30) of samples had DON levels that exceeded the recommended levels in Uruguay for swine and equines (1000 μg/kg); 10% (*n* = 11), the levels for cattle consumption (2000 μg/kg); and 3% (*n* = 3), the levels for ruminants, sheep and poultry (5000 μg/kg) [32]. No sample exceeded the Uruguayan levels for raw materials destined for the production of animal feed or the FDA levels for grains destined for ruminants and chickens (10,000 μg/kg) [29,32]. Zearalenone was the most dominant mycotoxin and occurred at high levels and incidence rates, with 64% (*n* = 72) of the samples exceeding the recommended levels in Uruguay of 250 μg/kg [32]. Although there are very few published works on ZEN contamination in sorghum worldwide, our results are similar to previous reports which positioned ZEN as the most frequent mycotoxin in sorghum [33,34,35]. High concentrations of DON and ZEN in our country could be explained by the fact that *F. graminearum s.s.* was the main contaminant species for sorghum. In this sense, we found a positive correlation between the incidence of *F. graminearum* and DON (*r* = 0.521, *p* < 0.001) and ZEN (*r* = 0.406, *p* < 0.001). In addition, climatic conditions, agricultural practices and the sorghum varieties used in Uruguay could also explain the levels of mycotoxins found.

In Uruguay, chemical protection for the control of *Fusarium* grain mold in sorghum is still not widely used, and little is known about the activity of fungicides used on crops, especially against FFSC. Therefore, this study provided information on fungicide sensitivity to two of the main toxigenic *Fusarium* species associated with sorghum crops. Metconazole was the fungicide that showed the best performance against *F. proliferatum* and *F. graminearum*, while epoxiconazole showed a lower effect compared to the other fungicides. In this sense, few studies have investigated the sensitivity of *Fusarium* spp. to epoxiconazole and particularly have been carried out with *F. graminearum* isolated from wheat. The sensitivity of *F. proliferatum* was similar to that reported by other authors in other geographical regions [36,37,38]. A single isolate of *F. proliferatum* exhibited EC50 values of 2.18 mg/L for metconazole, suggesting reduced sensitivity compared with the other isolates collected. However, except for this isolate, the sensitivity to metconazole was higher than those detected for the other fungicides. Regarding *F. graminearum s.s.* sensitivity, metconazole was the most effective fungicide in inhibiting the growth of *F. graminearum s.s.* isolates with EC50 values lower than 0.97 mg/L. However, the mean EC50 values found for epoxiconazole (1.97 mg/L) and tebuconazole (1.06 mg/L) were higher than those reported by other authors. For epoxiconazole, reported sensitivities in wheat from China ranged from 0.019 to 0.93 mg/L for isolates of *F. graminearum* [39]. In our case, 55% of the isolates had EC50 values higher than 0.93 mg/L, which was the highest value reported for Chinese isolates; moreover, we found one isolate that had an EC50 value of 15.29 mg/L. In the case of sensitivity to tebuconazole, our study showed a mean EC50 value (1.06 mg/L) higher than 0.891 mg/L and 0.29 mg/L, which were the mean values reported in other studies for isolates of *F. graminearum s.s*. from barley and wheat in Uruguay, respectively [17,19]. Sensitivity surely increases as years go by in relation to numerous exposures, especially to control *Fusarium* head blight. Moreover, our results agree with those obtained from wheat isolates in Brazil, China, Croatia and the United States [40,41,42,43]. Additionally, our findings were similar to previous reports that the EC50 values of isolates for metconazole were lower than the EC50 values of the same isolates for tebuconazole and the other fungicides [44]. To our knowledge, this is the first fungicide sensitivity analysis of different classes of fungicides for *F. proliferatum* and *F. graminearum* isolates from sorghum and constitutes an important starting point for defining management practices to control *Fusarium* grain mold in this crop. Further studies are needed to investigate the effectiveness of fungicide applications in vivo and the genetic basis for the reduced sensitivity of *Fusarium* species to epoxiconazole and tebuconazole.

## 4. Conclusions

Sorghum in Uruguay is exposed to colonization by a wide variety of toxigenic *Fusarium* species that could cause grain contamination with different mycotoxins, mainly from ZEN. These findings indicate the need to establish monitoring programs for mycotoxin levels in sorghum to ensure animal health and implement strategies to minimize fungal infection in the field. In addition to this, metconazole was found to be the most effective fungicide for control of the main *Fusarium* species analyzed.

## 5. Materials and Methods

### 5.1. Sorghum Samples

**Figure 4:**
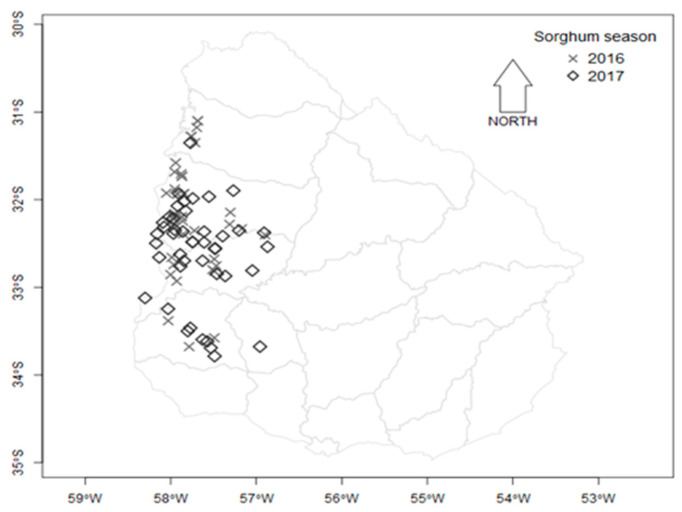
Map depicting the location of departments in Uruguay where sorghum samples were harvested in two sorghum growing seasons, 2016 and 2017.

A total of 167 sorghum samples were collected during two harvest seasons from fields in the main sorghum cultivation area of Uruguay (Figure 4). Ninety-five sorghum samples from the 2016 harvest season and 72 samples from the 2017 harvest season were analyzed. Samples were collected according to the ISO 24333: cereals and cereal products - sampling. Each 2 kg grain sample was divided into two subsamples (1 kg each), one for mycological analysis and the other for mycotoxin analysis.

### 5.2. Fusarium Isolation

From each sample, 100 grains were taken and surface disinfected by placing them in 0.4% hypochlorite for 2 min, rinsing with sterile distilled water and drying on sterile filter paper. Then, they were placed in Petri dishes, 10 grains per plate, with potato dextrose agar (PDA) culture medium (Oxoid) and incubated at 25 ± 2 °C for 5 days. Monosporic cultures were obtained from colonies identified morphologically as *Fusarium* spp. according to Leslie and Summerell [45]. A subset of representative isolates from different morphologically identified *Fusarium* species was selected for molecular identification, 223 isolates from the 2016 harvest and 221 isolates from the 2017 harvest. The isolates were stored at −80 °C in the fungal collection of the Sección Micología, Facultad de Ciencias, Universidad de la República.

### 5.3. Molecular Identification

Genomic DNA was extracted using the cetyltrimethylammonium bromide (2% CTAB) method [45].

Transcription elongation factor gene (*TEF 1-α*) RFLP analysis was performed to identify *Fusarium graminearum sensu stricto* (*F. graminearum s.s*) from the remaining species within FGSC with a protocol previously described [46]. A fragment of the *TEF 1-α* gene was amplified by PCR with primers EF-1 and EF-2 [47]. The PCR cycling parameters used for the amplification were as follows: an initial denaturation cycle at 95 °C for 3 min; 35 cycles of denaturation (1 min at 95 °C), hybridization (1 min at 55 °C) and extension (1:30 min at 72 °C); a final extension cycle of 10 min at 72 °C and 5 min at 4 °C. TEF 1-α amplicons were digested for 45 min at 37 °C with restriction enzyme *BsaHI*. RFPL patterns were visualized in a 1.5% agarose gel containing GreenSafe Premium (NZYTech, Lisboa, Portugal). Fragment sizes were estimated by comparison with a DNA standard marker GeneRuler 1 Kb DNA ladder (Thermo Scientific, Waltham, MA, USA).

For the other *Fusarium* species, the *TEF 1-α* gene was amplified in the same way as previously described, and PCR products were purified and sequenced by Macrogen Inc., Republic of Korea. Consensus sequences were obtained using the SeqMan software (Lasergene, Madison, WI, USA) and compared to GenBank database sequences using the Basic Local Alignment Search Tool (BLAST) and to the Fusarium MLST website (https://fusarium.mycobank.org) of CBS-KNAW Fungal Biodiversity Centre. The generated sequences were deposited in the GenBank database (Supplementary Table S1).

The sequences of FFSC were aligned with reference strain sequences from FFSC using the MUSCLE algorithm [48] with Mega version 11 [49]. A phylogenetic tree was inferred by using the maximum likelihood method, using Kimura’s 2-parameter model (G + I) and the Tree-Bisection-Regrafting (TBR) algorithm [50]. *Fusarium oxysporum* strain CBS 132475 and *Fusarium inflexum* NRRL 20433 were used as an outgroup. The analyses were conducted with 1000 bootstrap replicates.

### 5.4. Trichothecene Genotype Determination

Three different PCR assays were used to determine the trichothecene genotypes of 93 isolates of *F. graminearum s.s.* First, two multiplex assays targeted a portion of trichothecene biosynthesis genes *Tri3* and *Tri12* to determine NIV, 15-ADON and 3-ADON genotypes, while the third assay targeted portions of the *Tri13* gene to determine DON genotypes [51,52]. PCR for *Tri3* and *Tri12* consisted of an initial denaturation of 2 min at 94 °C, followed by 25 cycles of 30 s at 94 °C, 30 s at 52 °C and 1 min at 72 °C. PCR amplification of *Tri13* consisted of an initial denaturation of 2 min at 94 °C, followed by 35 cycles of 30 s at 94 °C, 30 s at 65 °C and 30 s at 72 °C. The specific bands for each amplification were resolved on 1.5% agarose gels containing GreenSafe Premium (NZYTech) and scored by size in comparison to a 100 bp DNA size ladder (Invitrogen Life Technologies, Carlsbad, CA, USA).

### 5.5. Fusarium Mycotoxin Production

The ability of 93 isolates of *F. graminearum* s.s and 55 isolates of *F. proliferatum* to produce mycotoxin was analyzed, as they were the most frequent species. For mycotoxin production assays, 25 g of rice grains was used for DON, NIV and ZEN or 20 g of rice was used for FB1 and FB2; rice grains were placed in bags with 10 mL of distilled water, and then autoclaved twice at 121 °C for 30 min. Autoclaved rice bags were inoculated with 3 mycelium plugs taken from pure cultures of each selected isolate and incubated for 28 days at 25 °C in the dark. After this time, the contents of the bags were dried at 50 °C, finely ground with a laboratory blender and stored at −20 °C until toxin analysis. DON and NIV were detected using a method described by Reynoso et al., and ZEN and FBs were detected based on AOAC 985.18 and AOAC: 995.15 methods, respectively [53,54]. All toxins were analyzed using a high-performance liquid chromatography (HPLC) instrument connected to a reversed-phase C18 column (150 mm × 4.6 mm i.d., 5 µm particle size; Nucleodur, Macherey-Nagel, Düren, Germany) connected to a Security Guard pre-column (8 mm × 4 mm i.d., 5 µm particle size; Nucleodur, Macherey-Nagel, Düren, Germany). The HPLC system consisted of a Shimadzu LC-10ADvp pump. For DON and NIV, the HPLC system was coupled with a Shimadzu SPD photodiode array detector, while for ZEN and FBs, an RF-10Axl fluorescence detector was used. Toxin production was determined by comparison with external standards supplied by Trilogy Analytical Laboratory Inc., Washington, MO, USA.

### 5.6. Determination of Mycotoxins in Sorghum

Fifty-five representative samples of sorghum grain from the 2016 harvest and 57 samples from the 2017 harvest were selected for mycotoxin determination according to AOAC methods [54]. All toxins were determined using an HPLC instrument connected to a reversed-phase Gemini C18 (4.60 mm × 150 mm, 5 μm) analytical column (Phenomenex, Torrance, CA, USA). The HPLC system consisted of a Waters 1525 pump, Waters 717 Injector and Waters TCM oven. For FBs and ZEN, the HPLC system was coupled with a Waters 2475 Fluorescence detector, while for DON and NIV, a Waters 2996 photodiode array detector was used. For FB analysis, the excitation and emission wavelengths were set at 335 and 440 nm, respectively. The mobile phase used was methanol–NaH_2_PO_4_ 0.1 M (77:23 *v/v*) pH = 3.3, at a flow rate of 1.0 mL/min and at 25 °C. For ZEN analysis, the excitation and emission wavelengths were set at 270 and 465 nm, respectively. The mobile phase was methanol–orthophosphoric acid 0.01 M (58:42 *v/v*), the flow rate was 1.0 mL/min at 25 °C. For DON and NIV analysis, the mobile phase was water–methanol (86:14 *v/v*), at a flow of 1.0 mL/min and at 30 °C. Standards of DON, NIV, FB1, FB2 and ZEN from Trilogy (Washington, MO, USA) were used. The accuracy of the method was evaluated through studies of accuracy and precision by using sorghum grains fortified with each toxin (DON, NIV, ZEN, FB1 or FB2 as appropriate). For the accuracy of the method, replicates of certified samples were performed, and the relative standard deviation was calculated between them. The percentage of recovery of the method was 93% for DON, 87% for NIV, 76% for FB1, 77% for FB2 and 77% for ZEN.

### 5.7. Fungicide Sensitivity Assay

The sensitivity of *F. proliferatum* and *F. graminearum s.s.* isolates were evaluated against two different chemical classes of fungicides, triazoles and benzimidazoles. Fungicides belonging to triazoles were metconazole, tebuconazole and epoxiconazole, while carbendazim belongs to the benzimidazole class. The assay was performed on a total of 20 isolates per species, 10 isolates from each crop season (2016 and 2017). Fungicides were diluted in dimethylsulfoxide (DMSO) and incorporated into PDA to achieve concentrations of 0.05, 0.25, 0.50, 0.75, 1.5, 3, 6 and 10 mg/L. Then, each PDA plate was inoculated with a mycelial plug in the center. *Fusarium proliferatum* isolate plates were incubated for 7 days at 25 °C in darkness, and *F. graminearum s.s.* strains were incubated for 5 days under the same conditions. Three replicates of each fungicide concentration per isolate were performed.

Evaluation was performed based on the radial growth on PDA containing different concentrations of each fungicide, compared to control plates (PDA + DMSO) [55,56]. The effective concentration of fungicides leading to a 50% inhibition (EC50) of mycelial growth of each strain was determined. The EC50 was calculated based on Probit analysis [57]. Analysis of variance (ANOVA) of the EC50 values was conducted to determine differences in sensitivity for each fungicide. Means were compared using Fisher’s least significant difference (LSD) (α = 0.05). Statistical analysis was performed using SigmaStat Version 3.5.

## Figures and Tables

**Table 1:** Contamination by toxins in sorghum samples harvested during 2016 and 2017 in Uruguay.

	Total Mean (µg/kg) ± SD		Positive Samples (%)		Contamination in Positive Samples (µg/kg)			
					Mean ± SD	Range	Mean ± SD	Range
	2016	2017	2016	2017	2016		2017	
DON	1315 ± 1597	565 ± 567	88	93	1507 ± 1623	59–6071	619 ± 564	59–2133
NIV	nd *	4 ± 19	0	5	--	--	79 ± 36	50–120
ZEN	1453 ± 2194	752 ± 1297	97	90	1567 ± 2239	30–9280	841 ± 1344	30–6560
FB1	67 ± 244	58 ± 148	16	23	411 ± 493	80–1693	255 ± 217	80–753
FB2	5 ± 36	13 ± 98	2	2	--	80–270	--	80–742

nd *: not detected.

## Data Availability

Data will be made available on request.

## Supplementary Materials

The following supporting information can be downloaded at: https://www.mdpi.com/article/10.3390/toxins15080484/s1, Figure S1: Maximum likelihood tree inferred from the transcription elongation factor gene (*TEF 1-α*) sequences of species belonging to the *Fusarium fujikuroi* species complex (FFSC). Table S1: GenBank accession numbers of the FFSC used in phylogenetic analysis.
