# Supplementary material for: Fusarium Species and Mycotoxins Associated with Sorghum Grains in Uruguay
Source: Toxins (Basel). 2023 Jul 31;15(8):484. doi: 10.3390/toxins15080484 (PMC10467058; doi:10.3390/toxins15080484)
Supplement: Supplementary file 1 [file toxins-15-00484-s001.zip › toxins-2494488-supplementary.pdf]

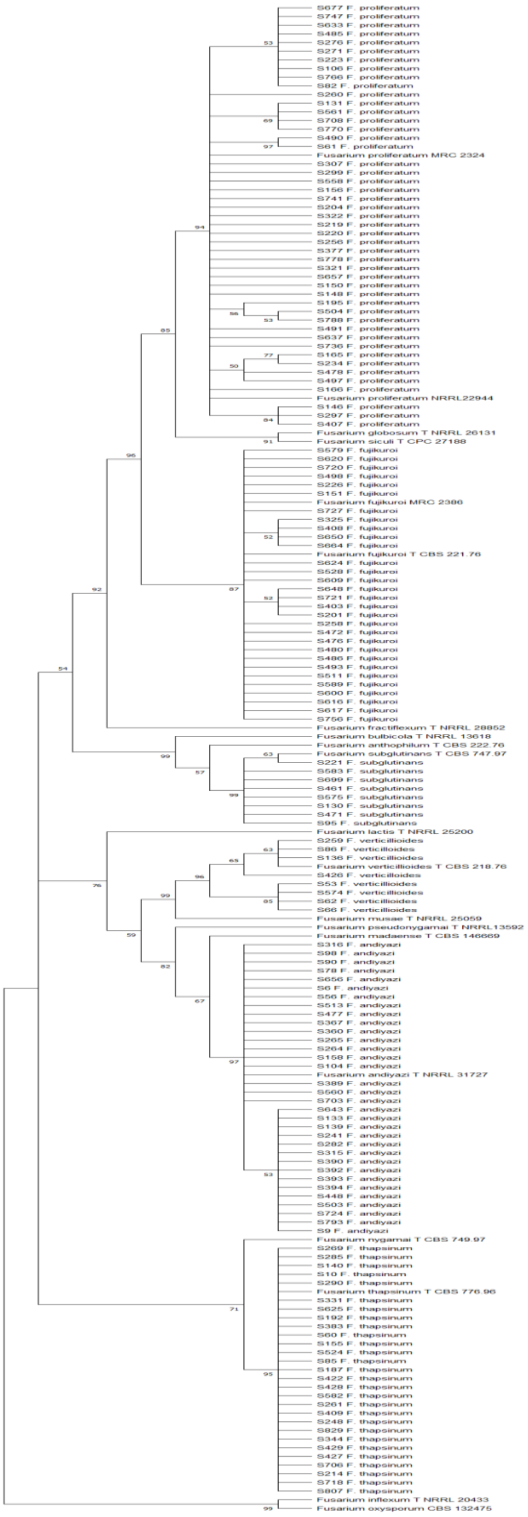

Figure S1: Maximum likelihood tree inferred from the transcription elongation factor gene (*TEF 1-α*) sequences of species belonging to the Fusarium fujikuroi species complex (FFSC).

Table S1: GenBank accession numbers of the FFSC used in phylogenetic analysis.

Species	Collection	TEF1a	Species	Collection	TEF1a
F. proliferatum	MRC 2324	MH582344	***F. fujikuroi***	MRC 2386	MH582339
	NRRL 22944	AF160280		CBS 221.76	MN534010
	S131	OQ702205		S201	OQ702134
	S148	OQ702206		S403	OQ702135
	S234	OQ702215		S408	OQ702136
	S256	OQ702216		S472	OQ702137
	S260	OQ702217		S498	OQ702138
	S271	OQ702218		S620	OQ702139
	S276	OQ702219		S624	OQ702140
	S297	OQ702220		S648	OQ702141
	S307	OQ702221		S650	OQ702142
	S321	OQ702222		S664	OQ702143
	S322	OQ702223		S151	OQ702144
	S377	OQ702224		S226	OQ702145
	S407	OQ702225		S258	OQ702146
	S478	OQ702226		S325	OQ702147
	S485	OQ702227		S476	OQ702148
	S491	OQ702228		S480	OQ702149
	S497	OQ702229		S486	OQ702150
	S504	OQ702230		S493	OQ702151
	S633	OQ702231		S511	OQ702152
	S637	OQ702232		S528	OQ702153
	S657	OQ702233		S579	OQ702154
	S677	OQ702234		S589	OQ702155
	S708	OQ702235		S600	OQ702156
	S736	OQ702236		S609	OQ702157
	S741	OQ702237		S616	OQ702158
	S747	OQ702238		S617	OQ702159
	S766	OQ702239		S720	OQ702160
	S770	OQ702240		S721	OQ702161
	S778	OQ702241		S727	OQ702162
	S788	OQ702242		S756	OQ702163
	S82	OQ702243	***F. subglutinans***	CBS 747.97	MW402150
	S61	OQ702244		S130	OQ702172
	S490	OQ702245		S221	OQ702173
	S146	OQ702246		S461	OQ702174
	S299	OQ702247		S471	OQ702175
	S558	OQ702248		S575	OQ702176
	S204	OQ702249		S583	OQ702177
	S561	OQ702250		S95	OQ702178

Species	Collection	TEF1a	Species	Collection	TEF1a
F. thapsinum	CBS 776.96	MN534044	***F. andiyazi***	S392	OQ656390
	S10	OQ702179		S393	OQ656391
	S140	OQ702180		S394	OQ656392
	S155	OQ702181		S448	OQ656393
	S187	OQ702182		S477	OQ656394

	S248	OQ702183		S503	OQ656395
	S261	OQ702184		S513	OQ656396
	S269	OQ702185		S56	OQ656397
	S285	OQ702186		S560	OQ656398
	S290	OQ702187		S6	OQ656399
	S331	OQ702188		S643	OQ656400
	S344	OQ702189		S656	OQ656401
	S383	OQ702190		S703	OQ656402
	S422	OQ702191		S724	OQ656403
	S427	OQ702192		S78	OQ656404
	S524	OQ702193		S793	OQ656405
	S582	OQ702194		S9	OQ656406
	S60	OQ702195		S90	OQ656407
	S625	OQ702196		S98	OQ656408
	S214	OQ702197		S264	OQ702130
	S409	OQ702198		S316	OQ702131
	S428	OQ702199		S389	OQ702132
	S429	OQ702200		S390	OQ702133
	S706	OQ702201	***F. verticillioides***	CBS 218.76	MW402113
	S807	OQ702202		S86	OQ702164
	S829	OQ702203		S66	OQ702166
	S85	OQ702204		S62	OQ702167
	S192	OQ702251		S574	OQ702168
	S718	OQ702252		S53	OQ702169
F. andiyazi	NRRL 31727	MN193854		S259	OQ702170
	S104	OQ656380		S136	OQ702171
	S133	OQ656381	***F. globosum***	NRRL 26131	KF466417
	S139	OQ656382	***F. lactis***	NRRL 25200	MN193862
	S158	OQ656383	***F. madaense***	CBS 146669	MW402098
	S241	OQ656384	***F. musae***	NRRL 25059	FN552086
	S265	OQ656385	***F. nygamai***	CBS 749.97	MW402151
	S282	OQ656386	***F. siculi***	CPC 27188	LT746214
	S315	OQ656387	***F. anthophilum***	CBS 222.76	MW402114
	S360	OQ656388	***F. pseudonygamai***	NRRL13592	AF160263
	S367	OQ656389	***F. bulbicola***	NRRL 13618	KF466415
	S367	OQ656389	***F. fractiflexum***	NRRL 28852	AF160288
